# Epidemiological Characteristics of Atrial Fibrillation in Southern China: Results from the Guangzhou Heart Study

**DOI:** 10.1038/s41598-018-35928-w

**Published:** 2018-12-13

**Authors:** Hai Deng, Pi Guo, Murui Zheng, Jun Huang, Yumei Xue, Xianzhang Zhan, Feng Wang, Yang Liu, Xianhong Fang, Hongtao Liao, Wei Wei, Yuanhong Liang, Fangzhou Liu, Zili Liao, Yijing Feng, Shulin Wu

**Affiliations:** 1 Guangdong Cardiovascular Institute, Guangdong General Hospital, Guangdong Academy of Medical Science, Guangzhou, 510080 China; 2 Department of Preventive Medicine, Shantou University Medical College, Shantou, 515041 China; 3 Guangzhou Center for Disease Control and Prevention, Guangzhou, 510440 China; 4 Department of Geriatrics, Guangdong General Hospital, Institute of Geriatrics, Guangdong Academy of Medical Sciences, Guangzhou, 510080 China; 5 Johns Hopkins Bloomberg School of Public Health, Baltimore, Maryland 21205 USA

## Abstract

Precise prevalence of atrial fibrillation (AF) and the associated risk factors in southern China are rarely reported. This large population-based follow-up study, the Guangzhou Heart Study, was conducted from 2015 to 2017 to fill up this gap. Permanent residents aged 35 years and above in Guangzhou city were enrolled and demographic factors of participants were collected by a structured questionnaire. Examinations of physical, electrocardiographic and biochemical indicators were performed following a standard operation procedure designed prior to the field investigation. Descriptive statistics were used to evaluate basic characteristics of the study participants, and multivariate logistic regression model was performed to assess the AF prevalence-related factors. The detailed study design, the baseline characteristics and the prevalence of AF were reported here. In total, 12,013 residents were enrolled, and the percentage of participants from rural and urban areas was 53.92% and 46.08%, respectively. In total, 90.57% participants aged 40–79 years old and the proportion of women was more than men (64.98% *vs*. 35.02%). Overall, the prevalence of AF among the participants was 1.46%. Increasing age, male sex and widowed marital status were associated with higher AF prevalence (*P*-value < 0.05). The prevalence of AF increased with age and climbed to approximately 5% in residents aged 80 years and over. Residents with abnormal higher blood level of total cholesterol tended to have a lower AF prevalence but a higher prevalence of AF was observed in female participants with lower level of high density lipoprotein cholesterol land higher level uric acid (all *P*-value < 0.05). Personal illness such as hypertension, diabetes mellitus, dyslipidemia, myocardial infarction, heart failure, stroke and transient ischemic were significantly linked to the attack of AF (all *P*-value < 0.05). This study will be rich resource for investigating environmental exposure and individual genetic diathesis of AF and other common cardiovascular diseases in Chinese population.

## Introduction

Atrial fibrillation (AF) is a major public health burden worldwide, and it is the most common arrhythmia in clinical practice and can result in significant morbidity, including thromboembolic events, increased risk of stroke, and a 1.5- to 2-fold elevation in mortality, independent of other risk factors^1–3^. It reduces the quality of life and causes a significant economic burden on patients^4,5^.

Some previous studies from the Euro and the US have determined prevalence and lifetime risks of AF. A study from UK presented the prevalence of all age “active” AF rose from 0.78% to 1.31% in men and from 0.79% to 1.15% in women during 1994–2003^6^. The Framingham Heart Study reported a prevalence of 1.7% AF without comorbidities in the entire cohort and an annual incidence of 0.5 per 1000 person-year^7^. Lifetime risks for AF were 26.0% and 23.0% at the age of 40 years among men and women, respectively, and did not change significantly with increasing index age because of rapid rise in AF incidence with advancing age^8^.

According to a recent review, the prevalence of AF is set to increase owing to widespread population aging, especially in rapidly developing countries such as China, Brazil, India, and Indonesia^9^. A Taiwanese provincial population-based study among more than 23 million people demonstrated a relative risk of 1.92 for AF among individuals with a first-degree relative affected by AF^10^. In addition, the Guangzhou Biobank Cohort Study identified both general and central obesity was related to increased risk of AF^11^. However, very few large-scale studies to date have investigated the prevalence and relevant risk factors of AF in southern China. We found that knowledge of AF in the Chinese population, especially the southern Chinese population, were limited. Therefore, we conducted a large population-based study, the Guangzhou Heart Study, to collect the baseline data from the permanent residents during the period from 2015 to 2017 in Guangzhou, the most densely-populated city in southern China, and then follow to examine their health status every 2 or 3 years. In this study, we initially reported the details about the study design, the main baseline characteristics of the participants, and the prevalence and relevant risk factors of AF of the study participants.

## Materials and Methods

### Survey design

**Figure 1:**
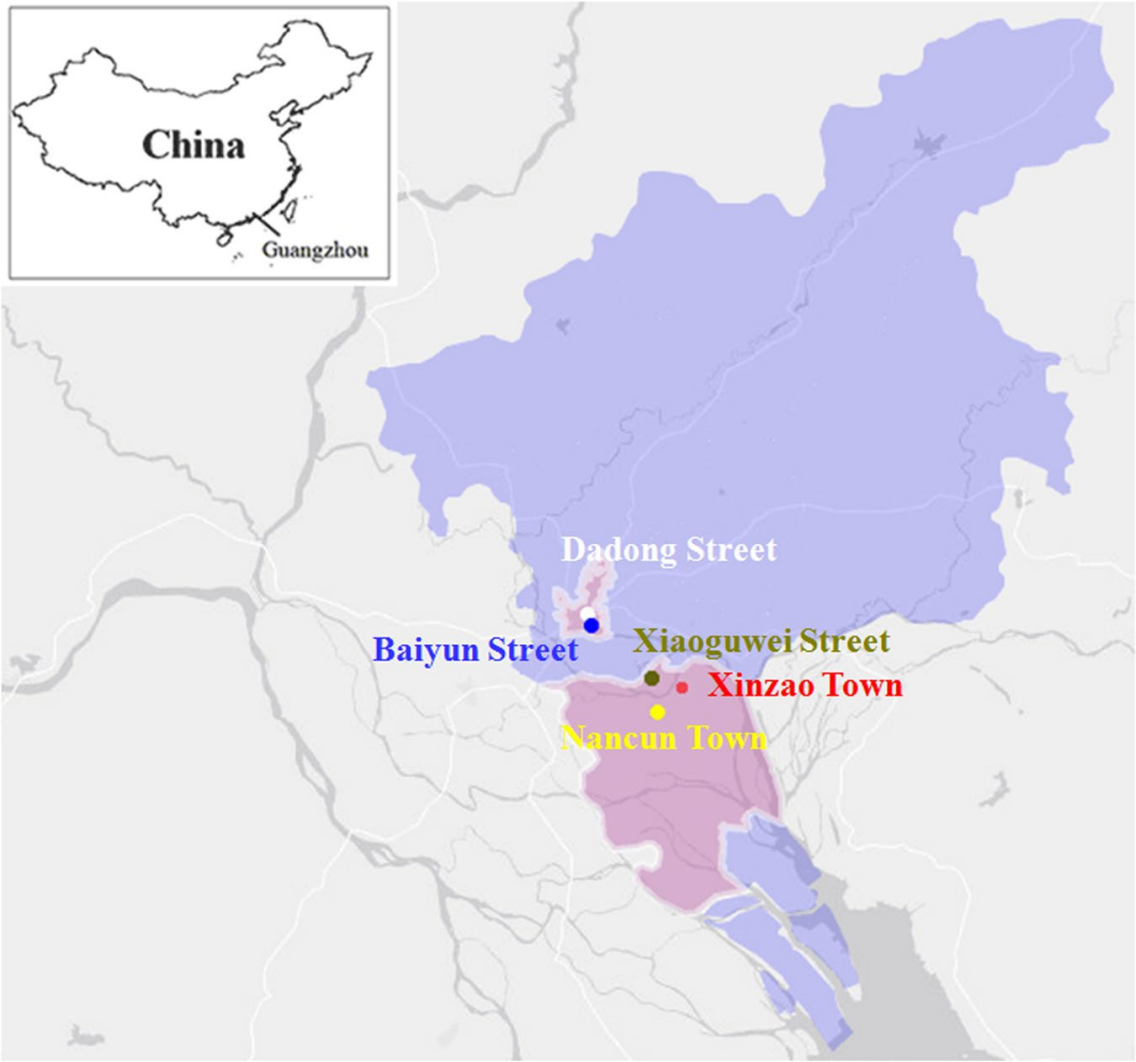
Geographical location of the study sites. Permanent residents aged 35 and above were recruited from the Yuexiu and Panyu districts in Guangzhou city, southernChina. Dadong and Baiyun streets from Yuexiu District, and Xiaoguwei Street, XinzaoTown and NancunTownfromPanyu District were selected as the study sites.

This population-based study, the Guangzhou Heart Study, took place in July 2015, and the baseline data were initially collected until August 2017 in Guangzhou. Randomized multistage cluster sampling was used to recruit permanent residents aged 35 and above from Yuexiu and Panyu districts in Guangzhou city. Of the two districts, two streets (Dadong Street and Baiyun Street) were randomly selected from Yuexiu District to stand for urban areas while one street and two towns (Xiaoguwei Street, Xinzao Town and Nancun Town) from Panyu District were chosen as rural areas. The geographical location of the study sites is shown in Fig. 1. The residents in the study sites were invited to participate in this study by 3-round mobilization via door-to-door visits or telephone appointments.

Prior to enrollment, the residents were informed of the details of this study and gave written informed consent which allows access to their medical tests and records. After informed consent was obtained, all participants were interviewed face-to-face by the trained field researchers who were received systematical training for questionnaire, physical measurements, and quality control to guarantee standardization of the investigation procedure. Regarding the electrocardiograph examination (ECG), 24-hour single lead ECG examination and ultrasonic cardiogram (UCG, only for AF patients and senior citizens aged 65 and above), these medical tests were conducted by the experienced professionals from Guangdong Cardiovascular Institute. In addition, all the devices regularly maintained and calibrated to ensure consistency of measurements. This study was approved by the Guangzhou Medical Ethics Committee of the Chinese Medical Association. All procedures performed in studies involving human participants were in accordance with the ethical standards of the 1964 Helsinki Declaration and its later amendments or comparable ethical standards.

### Study population and data collection

All the participants were strictly identified at the registry desk in every local community health center or village hospital according to the following inclusive and exclusive criterion. The inclusive criterion were: (1) Guangzhou permanent residents who were registered in the Guangzhou Household Register System; (2) 35 years old and above; (3) living in the selected communities for at least 6 months from the day they came to participate in the survey. The exclusive criterion were: (1) mental or cognitive disorders including dementia, disturbance of understanding, and deaf-mutters; (2) mobility difficulties including high paraplegia; (3) pregnant or lactating women; (4) malignant tumors under treatments; (5) floating people including those who rented the houses or apartments; (6) Guangzhou residents who not living in the selected communities for at least 6 months by the day they participant the survey; (7) non-Guangzhou residents; (8) non-responders during the 3-round mobilization.

At the baseline survey, a structured and interviewer-administered questionnaire was used to compile each participant’s demographic information and relevant epidemiological factors for AF. The standard questionnaire consisted of several domains related to general demographic and socioeconomic characteristics (including age, gender, career, marital status, education level, occupation and income, etc), personal history of disease (including hypertension, diabetes, coronary disease, and stroke, etc), family history of disease, lifestyle habits (including tea, coffee, cigarette and alcohol consumption, and physical activities, etc), medications and health-related habits, and evaluation of personal depression and anxiety. The physical measurement (neck, waist, and hips circumference, height, weight, blood pressure and heart rate) and body fat measurement (body mass index (BMI), basal metabolic rate, percentage of body fat and visceral fat index) were performed using standard instruments and protocols. The measurements were recorded twice and the mean of the two values were used for analysis. A range of blood tests were assessed according to standardized procedures, including routine tests, fasting blood-glucose (FBG) and lipids, renal and liver function examinations, C-reactive protein (CRP). All the blood samples were detected in a third-party medical laboratory and stored for further analysis. All the baseline data were entered into a pad-based dataset entry system. Response rate for participants who finished every step of the survey in this study was 41.16% in total (39.24% in Yuexiu District and 42.75% in Panyu District, respectively).

### Diagnosis of AF

Subjects were diagnosed with AF meeting any one criterion as follows: (1) ECG screening of subject shows AF pattern; (2) ECG screening of dose not find AF but subject has AF history with evidence such as past AF ECG, in-hospital diagnosis information or record of ablation or cardiac surgery; (3) 24 hours single-lead ECG record shows AF episodes. The criterion of diagnosis of AF followed the ACC/AHA/ESC 2016 guidelines^12^. ECG and single-lead 24 hours ECG monitor were performed by well-trained physician and AF diagnosis were determined by two specific electrophysiological experts.

### Quality control

There was three-round quality control (QC) for the baseline data in this study. In the first round (self-check QC), the interviewers checked their questionnaires after the interviews to fill in the missing information and correct the errors. In the second round (field data QC), paper questionnaires were collected to the data quality controllers after the questionnaire interviews identified information inconsistency (including survey ID, name, identity card, date of birth, etc), missing data and logistic errors. Inconsistent data were correctly matched while missing data and logistic errors were recollected by the correspondent interviewers calling back to the interviewees. If the logistic errors were due to the mistakes which the interviewers ticked the wrong items, they would be corrected by themselves. In the third round (background data QC), the data quality controllers mainly focused on the background data in the website where the data had been transmitted from pad-based system to determine the correctness of data entry. Mismatches and extreme values were identified and revised according to data recorded in the paper questionnaire. All the modified data were verified after the three-round QC.

### Statistical analysis

Descriptive statistics were used to evaluate basic characteristics of the study participants, and the χ^2^ test was performed to compare the categorical variables. The prevalence of AF according to the categories grouped by demographic characteristics including age, gender, height, BMI, nationality, marital status, educational level and career type was compared by the χ^2^ test. In addition, we compared the prevalence of AF according to the categories of participants’ blood biochemical factors including total cholesterol, triglyceride, high density lipoprotein cholesterol, low density lipoprotein cholesterol, uric acid, blood sugar and creatinine, using the χ^2^ test, for men and women participants, respectively. Finally, multivariate logistic regression models were developed to investigate the associations between the prevalence of AF and personal history of illness. The dependent variable in the models was the outcome of AF for each participant, and the risk of AF linked to personal history of hypertension, diabetes, myocardial infarction, heart failure, stroke, transient ischemic attack, syncope, congenital heart disease and valvular heart disease after controlling for age and gender were evaluated, respectively. The odds ratios (ORs) and corresponding 95% confidence intervals (95% CIs) were calculated to assess the associations.

SAS software version 9.3 (SAS Institute Inc.; Cary, NC) was used for the statistical analyses. All statistical tests were 2-sided, and a *P*-value < 0.05 was considered to be statistically significant.

## Results

### Basic characteristics of the study participants

Almost all the recruited participants completed the questionnaires and the blood samples collection, resulting in an eligible dataset involving 12,013 people after checking the validity of each questionnaire. Overall, the estimated prevalence of AF among the study participants in Guangzhou was 1.46%.

**Figure 2:**
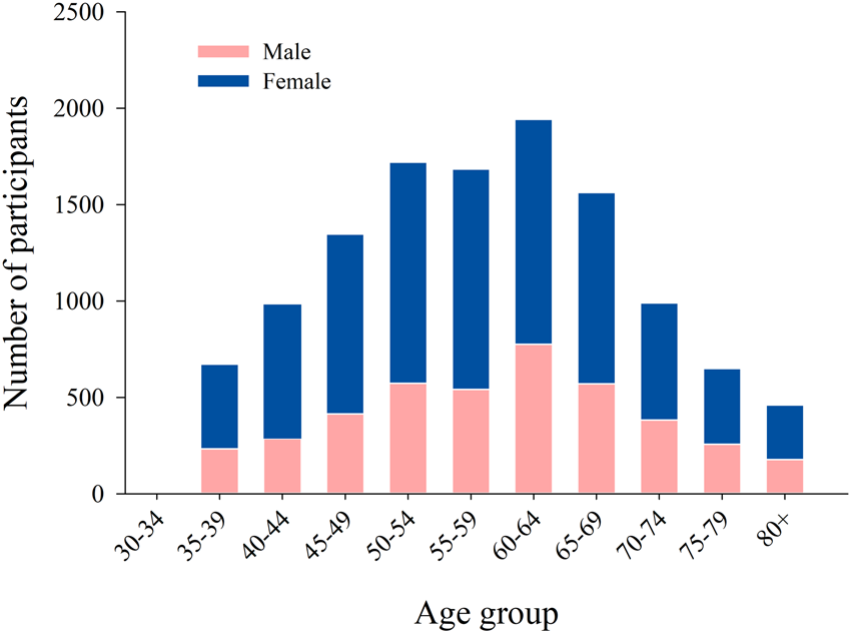
Age distribution by gender of the study participants in Guangzhou, southern China.

Table 1 shows the basic characteristics of the study participants. In this study, the percentage of participants from rural and urban areas was 53.92% and 46.08%, respectively. Among them, 90.57% participants enrolled were aged 40 to 79. The distribution of age of the entire study participants and that grouped by gender is shown in Table 1 and Fig. 2, respectively. The sex ratio (male *vs*. female) in our study was around 0.54:1. The participants less than 155 cm in height accounted for 37.52%, and the figure for those who were between 155 cm and 159 cm tall was nearly 21.9%. In addition, most of them had the BMI ranging from 22.5 kg/m^2^ to 30 kg/m^2^, accounting for around 58.6%. Overall, among the study participants, about 59.9% had an education level of middle school and above, and people who married made up the largest portion (82.3%). The most common type of occupation was transportation and equipment operators related personnel, account for 20.6%.

**Table 1:** Basic characteristics of the study participants in Guangzhou, southern China.

Characteristics	Category	Frequency	Percentage
Subject	Atrial fibrillation patients	175	1.46
	Non-atrial fibrillation patients	11838	98.54
Region	Rural	6478	53.92
	Urban	5535	46.08
Age (years)	30–39	673	5.60
	40–49	2329	19.39
	50–59	3405	28.34
	60–69	3506	29.19
	70–79	1640	13.65
	≥80	460	3.83
Gender	Men	4207	35.02
	Women	7806	64.98
Height (cm)	<155	4507	37.52
	155–159	2629	21.88
	160–164	2166	18.03
	≥165	2711	22.57
BMI (kg/m^2^)	<18.5	862	7.18
	18.5 to <22.5	3478	28.95
	22.5 to <25	3446	28.69
	25 to <30	3598	29.95
	≥30	629	5.24
Marital status	Married	9892	82.34
	Divorced	208	1.73
	Separated	34	0.28
	Widowed	1284	10.69
	Single	216	1.80
	Missing	379	3.15
Educational level	Illiteracy	1764	14.68
	Primary school	2672	22.24
	Junior middle school	2782	23.16
	High and vocational school	2860	23.81
	Junior College	993	8.27
	Undergraduate and above	563	4.69
	Missing	379	3.15
Occupation	Leader of enterprise unit	517	4.30
	Technical personnel	809	6.73
	Handle affairs personnel	1328	11.05
	Sales and service personnel	1733	14.43
	Agricultural, forestry, fishery and water conservancy production staff	1603	13.34
	Transportation and equipment operators related personnel	2474	20.59
	Army man	16	0.13
	Private owner	339	2.82
	Others	2815	23.43
	Missing	379	3.15

### Prevalence of AF based on different demographic characteristics

Table 2 shows the AF prevalence based on the selected demographic characteristics of the study participants. The study found rural residents had a comparable prevalence of AF with urban residents (1.33% *vs*. 1.61%). The prevalence was lowest among participants aged 40–49 (0.13%) and the highest prevalence was observed among those aged above 80 years (5.00%). The prevalence of AF differs by age group and gender, as shown in this study (*P*-value < 0.05). In fact, male residents had a significantly higher prevalence of AF (2.02%) than that (1.15%) of women.

**Table 2:** Prevalence of atrial fibrillation based on demographic characteristics.

Characteristics	Frequency	Prevalence (%)	*P*-value
Region			0.2011
Rural	6478	1.33	
Urban	5535	1.61	
Age (years)			<0.0001^§^
30–39	673	0.30	
40–49	2329	0.13	
50–59	3405	0.73	
60–69	3506	1.65	
70–79	1640	3.90	
≥80	460	5.00	
Gender			0.0002^§^
Men	4207	2.02	
Women	7806	1.15	
Height (cm)			0.0006^§^
<155	4507	1.11	
155–159	2629	1.79	
160–164	2166	0.97	
≥165	2711	2.10	
BMI (kg/m^2^)			0.2485
<18.5	862	1.74	
18.5 to <22.5	3478	1.27	
22.5 to <25	3446	1.22	
25 to <30	3598	1.72	
≥30	629	1.91	
Marital status			<0.0001^§^
Married	9892	1.23	
Divorced	208	0.96	
Separated	34	0.00	
Widowed	1284	3.19	
Single	216	0.00	
Educational level			0.0045^§^
Illiteracy	1764	2.27	
Primary school	2672	1.61	
Junior middle school	2782	1.15	
High and vocational school	2860	0.98	
Junior College	993	1.11	
Undergraduate and above	563	1.95	
Occupation			0.0003^§^
Leader of enterprise unit	517	1.93	
Technical personnel	809	1.24	
Handle affairs personnel	1328	1.96	
Sales and service personnel	1733	0.69	
Agricultural, forestry, fishery and water conservancy production staff	1603	2.50	
Transportation and equipment operators related personnel	2474	1.25	
Army man	16	6.25	
Private owner	339	0.88	
Others	2815	1.14	

^§^This symbol indicates *P*-value < 0.05 based on the χ^2^ test.

Our analysis revealed that among widowed people the AF prevalence was significantly higher than that among people with other marital status (*P*-value < 0.05). Regarding the factor of educational level, we found that participates with different educational level had different prevalence of AF (*P*-value < 0.05), suggesting that illiteracy goes together with the highest prevalence (2.27%). Similarly, we also identified that the AF prevalence differs by occupation type, and people served as army men had the highest incidence of AF (*P*-value < 0.05).

**Figure 3:**
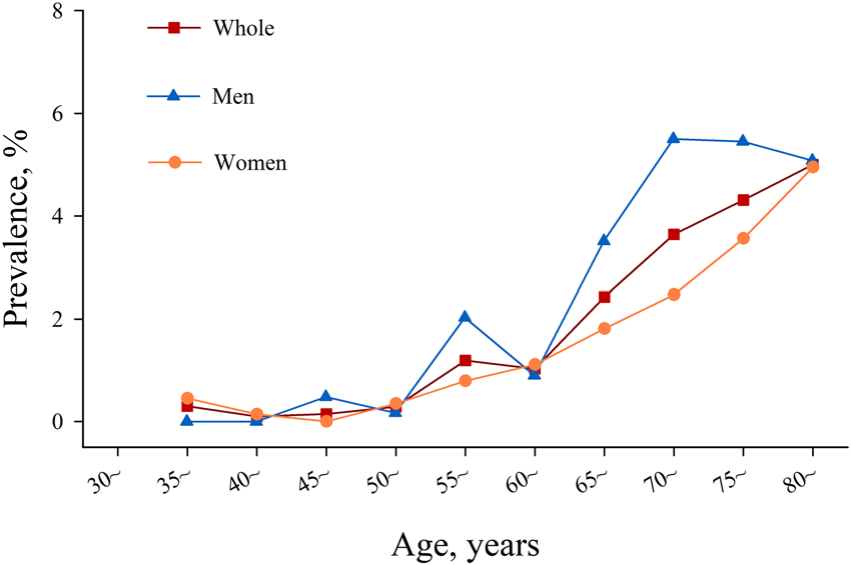
Prevalence of atrial fibrillation at various ages by gender. Three groups of people including the entire participants, men and women participants were analyzed.

We further estimated the prevalence of AF separated by age group for every 5 years among the entire, men and women participants, respectively (Fig. 3). It demonstrated that the prevalence of AF tended to be higher in men than in women, and the highest prevalence occurred among the people aged 80 and above.

### Prevalence of AF by personal blood biochemical factors and history of illness

Table 3 shows the differences in the prevalence of AF according to by the blood biochemical factors of participants by gender. We found that for both men and women, participants with elevated levels of blood sugar and creatinine tended to have higher prevalence of AF (*P*-value < 0.05) but lower prevalence with elevated levels of total cholesterol (*P*-value < 0.05). In particular, higher prevalence of AF appeared among women participants with low levels of high density lipoprotein cholesterol and high levels of uric acid than those with normal indicators (*P*-value < 0.05).

**Table 3:** Comparison of prevalence of atrial fibrillation according to participants’ blood biochemical factors.

Biochemical factors	Men			Women		
	Frequency	Prevalence (%)	*P*-value	Frequency	Prevalence (%)	*P*-value
Total cholesterol			0.0213^§^			0.0122^§^
Not -elevated	2039	2.45		2914	1.51	
Elevated	2059	1.46		4634	0.88	
Triglyceride			0.2445			0.8042
Not-elevated	3215	2.08		6377	1.11	
Elevated	883	1.47		1170	1.20	
High density lipoprotein cholesterol			0.5180			0.0091^§^
Not-elevated	3768	1.91		6505	1.00	
Elevated	330	2.42		1043	1.92	
Low density lipoprotein cholesterol			0.0873			0.0601
Not-elevated	3099	2.16		5245	1.28	
Elevated	999	1.30		2303	0.78	
Uric acid			0.1591			<0.0001^§^
Not-elevated	2163	1.66		4108	0.99	
Elevated	1935	2.27		3440	1.87	
Blood sugar			0.0146^§^			0.0088^§^
Not-elevated	3390	1.71		6369	1.15	
Elevated	708	3.11		1179	2.26	
Creatinine			0.0006^§^			<0.0001^§^
Not-elevated	3346	1.64		6836	0.91	
Elevated	861	3.48		970	2.89	

^§^This symbol indicates *P*-value < 0.05 based on the χ^2^ test.

Table 4 shows the associations between AF prevalence and participants’ personal history of illness. We found that the significantly increased risk for AF attack was more frequently seen in participants with personal history of hypertension (OR: 1.57, 95% CI: 1.10 to 2.12), diabetes (OR: 1.62, 95% CI: 1.09 to 2.41), dyslipidemia (OR: 1.65, 95% CI: 1.18 to 2.30), myocardial infarction (OR: 2.68, 95% CI: 1.54 to 4.66), hear failure (OR: 6.21, 95% CI: 3.38 to 11.42), stroke (OR: 2.54, 95% CI: 1.42 to 4.57), transient ischemic attack (OR: 3.30, 95% CI: 1.83 to 5.93).

**Table 4:** Associations between atrial fibrillation prevalence and individual history of illness.

Variables	Groups	Prevalence, %	Multivariate logistic	*P*-value
			OR (95% CI)	
Hypertension	No^†^	0.90	1	0.0121^‡^
	Yes	2.64	1.57 (1.10, 2.12)	
Diabetes	No^†^	1.25	1	0.0163^‡^
	Yes	3.09	1.62 (1.09, 2.41)	
Dyslipidemia	No^†^	1.19	1	0.0032^‡^
	Yes	2.34	1.65(1.18, 2.30)	
Myocardial infarction	No^†^	1.31	1	0.0005^‡^
	Yes	7.27	2.68 (1.54, 4.66)	
Heart failure	No^†^	1.31	1	<0.001^‡^
	Yes	11.38	6.21 (3.38, 11.42)	
Stroke	No^†^	1.32	1	0.0018^‡^
	Yes	7.00	2.54 (1.42, 4.57)	
Transient ischemic attack	No^†^	1.32	1	<0.001^‡^
	Yes	7.82	3.30 (1.83, 5.93)	
Syncope	No^†^	1.34	1	0.0576
	Yes	2.30	1.56 (0.99, 2.48)	

^†^The reference category. ^‡^This symbol indicates that the category is significantly different from the reference category (*P*-value < 0.05).

## Discussion

This large population-based study, the Guangzhou Heart Study, was conducted to investigate the epidemiological characteristics and relevant factors of AF among permanent residents in Guangzhou city, the most densely-populated city in southern China. In this present study, we initially reported the details about the study design, the main baseline characteristics and the prevalence of AF. We summarized the main findings as follows: first, the estimated prevalence of Guangzhou residents was 14.6 per 1000 people with a tendency to increase with age; second, the risk of AF occurrence was likely related to gender, marital status, education, occupation, abnormal blood fat and personal history of illness. More results from the Guangzhou Heart Study will be successively reported in future studies.

According to official statistics of population of Guangzhou in 2017, there were about 5.8 million persons aged 35 and above, which meant that at least 85 thousand adults lived in this city suffered from the disease of AF. The incidence of AF in population aged below 40 was just 0.3% while increased sharply to 1.65% in those aged 60–69 years and 5.0% in people aged 80 years and above. With the prevalent trend in the elderly, we believed that AF cases distributed in people aged 60 years and above mainly contributed to the overall prevalence. According to the Framingham Study, lifetime risk for the development of AF was 1 in 4 men or women aged 40 years or older^8^. Hence, it is reasonable that the AF prevalence of Guangzhou residents may grow up following the population aging.

The global prevalence of AF surged owing to research regions, races, ethnics as well as economic differences^13^. In Australia, Europe, and the USA the estimated prevalence of AF in adults ranged from 1–4%, and rose to over 13% of individuals aged >80 years^9^. Outside the Europe and North American, reported prevalence of AF varied from countries, with different ranges from 0.1–4% in community-based studies^14^. Differences were observed in Asia either. The prevalence of AF of South Korea^15^ in men and women aged 30–39 years was 0.08% and 0.03%, respectively, increasing to 2.35% and 1.71%, respectively, in those aged ≥60 years according to its National Health Insurance Service-National Sample Cohort database. Resent reported data demonstrated that the prevalence of AF in Hong Kong is 1.8% and age, sex, different anthropometric parameters and cardiovascular comorbid conditions were found as independent risk factors of AF^16^. We can find different prevalence even in Chinese cohort. Yang *et al*. reported a 1.07% in residents aged ≥40 years of a south-northern community^17^ and Li *et al*. presented a 1.57% in residents aged ≥40 years enrolled from 31 provinces^18^. Our result is similar to that of Li’s^18^ and the different age standard for enrollment should be a reason of the minor difference.

A previous survey^11^ based on a small population has reported the AF prevalence of Guangzhou citizens, and found that the prevalence was 0.8% in aged 50 and older citizens. However, in this large population-based epidemiological study involving 12,013 participants, the prevalence of AF is estimated as 1.46%. Although we conducted a similar community-based and cross-sectional designed study, we performed cluster sampling technique which considered the characteristics of population distribution and the diagnosis of AF was determined by two experts with standard QC which ensured the accuracy of prevalence estimates of AF. In addition, we also found that the estimated prevalence of AF varied across different groups of age, which was 1.46% in the people aged 35 years and older.

Just like many other study^15,16,19,20^, we found male and female with increased blood uric acid and decreased HDL is related to AF incidence in our study. Hyperuricemia is observed in more than 50% residents in our study. Hyperuricemia was reported as an independent risk factor of AF^21,22^. Strong link with prevalence of persistent, longstanding even paroxysmal AF was observed in patients with type 2 mellitus diabetes which is another popular kind of metabolic diseases^23,24^. But in our study, only women with hyperuricemia were related to AF occurrence. Gender specific relationship between hyperuricemia and AF was also reported elsewhere^25,26^ but the mechanism is not clear yet.

Dyslipidemia is common according the result of our study. Incidence of elevating total cholesterol (TC) and decreasing high density lipoprotein (HDL) was 50.2%, 23.4% in men, and 61.4%, 30.5% in female respectively. Association of elevated TC and decreased HDL in female with AF occurrence was observed in our study while TC was reported inversely associated with new onset AF in another Chinese cohort^27^.

We found that personal history of hypertension, type 2 diabetes mellitus, dyslipidemia, coronary artery disease and heart failure were associated with high prevalence of AF in our study which was similar with a previous report^28^. Accumulated evidence showed that obesity is related to AF incidence and its arrhythmia outcome^28,29^ and strongly linked to prevalence of hypertension, diabetes mellitus, dyslipidemia and other cardiovascular disease^30,31^. Association between BMI and AF was not found in our study. The cross-sectional design of the study and BMI was not analyzed in subgroups as underweight, normal weight, over weight and obesity may mainly contribute to the different result. The impact of BMI in AF will be validated by further analysis and future follow-up study.

Socioeconomic factors were related to the incidence and outcome of AF or other vascular disease. In this study, we found residents who were in widowed marital status, served as army man, and accepted high or illiteracy education had higher risk of AF. Result of a survey on AF Swedish cohort^32^ aged 40 years and above showed that, higher educational level was associated with a reduced mortality while divorced and unmarried men had an increased risk of death. In another research, middle-age Swedish suffer more stoke being unmarried^33^. We didn’t find any other report about widow marital status and its impact in AF incidence. The reason of the result should be complicated which may be related to other socioeconomic situation and emotion status.

Besides, although this large population-based epidemiological study to reveal the prevalence and relevant risk factors of AF in Guangzhou, at the same time we should also pay attention to the limitations of this study. First, incidence of AF and its risk factors may be associated with local food intaking habits, living surrounding, health care condition and economic situation. Regional incidence and related risk factors of AF study should be further analyzed to reveal the mechanisms of such differences and provide protective advice for the disease. Second, we did not identify independent predictors of arrhythmia progression, stoke, cardiovascular death or all cause death of residents with AF. Continuous studies should be performed to investigate this issue in the future. Third, the difference in the prevalence and related factors of AF in urban and rural areas is not yet fully understood, which also need to be further studied and explored.

## Conclusions

The estimated prevalence of AF in Guangzhou was 1.46% and related to age, gender, socioeconomic situation, abnormal blood biochemical index and personal illness. Results of this epidemiological study on AF prevalence and associated factors in Guangzhou paid a special attention to the characteristics of AF in southern China. This established large population-based study will be rich resource for investigating environmental exposure and individual genetic diathesis of AF and other common cardiovascular diseases in the Chinese population. The information generated will be of general relevance to better understand cardiovascular diseases in China.
